# Five-Year Sales Trends of Osteoporosis Medications in Korea: A Market Analysis Based on IMS Health Sales Audit Data (2018–2023)

**DOI:** 10.3390/medicina61050805

**Published:** 2025-04-26

**Authors:** Jung Yoon Park, Youn-Jee Chung, Mee-Ran Kim, Jae-Yen Song

**Affiliations:** Division of Reproductive Endocrinology, Department of Obstetrics and Gynecology, Seoul St. Mary’s Hospital, College of Medicine, The Catholic University of Korea, 222 Banpo-daero, Seocho-gu, Seoul 06591, Republic of Korea; aurorix86@naver.com (J.Y.P.); porshe80@catholic.ac.kr (Y.-J.C.); drmrkim@gmail.com (M.-R.K.)

**Keywords:** osteoporosis, romosozumab, denosumab, hormone replacement therapy, menopause, marketing, retrospective studies

## Abstract

*Background and Objectives*: Osteoporosis is a common chronic condition after menopause that increases the risk of fractures. In South Korea, the prevalence of osteoporosis among adults aged 50 and older is 22.4%, with 94.4% of treated patients being women, highlighting its significant impact on postmenopausal health. In this study, we examine the sales trends of osteoporosis medications in Korea from 2018 to 2023 to understand current usage patterns and market dynamics. *Materials and Methods*: This study is a retrospective analysis based on pre-recorded sales data from Intercontinental Marketing Services (IMS). Data covering a five-year period (2018–2023) were analyzed to examine the sales trends of osteoporosis medications, including bisphosphonates, selective estrogen receptor modulators (SERMs), parathyroid hormone analogs, denosumab, romosozumab, and others. Romosozumab, approved in November 2019, was included in the analysis. Given the nature of this study, no direct patient data or clinical interventions were involved. *Results*: The total market size for osteoporosis medications in South Korea reached USD 285.42 million in 2023, reflecting a 15.3% increase from 2022. Bisphosphonates, previously the dominant therapy, experienced an 11% decline in market share over five years. Meanwhile, denosumab, a receptor activator of the nuclear factor-κB ligand inhibitor, showed a remarkable growth rate of 957.6% from 2018 to 2023, surpassing bisphosphonates in their market share. Romosozumab, a newly introduced anabolic agent, accounted for 7.4% of the market, with sales increasing by 59% in 2023. *Conclusions*: This analysis revealed major shifts in treatment preferences, with newer drugs like denosumab and romosozumab gaining prominence over traditional bisphosphonates. These trends highlight the increasing clinical adoption of anabolic agents for high-risk patients and the impact of expanded reimbursement policies on osteoporosis management. Given the increasing use of advanced therapies, it is essential to monitor treatment access, patient adherence, and long-term clinical outcomes. Understanding these sales trends can aid healthcare professionals and policymakers in optimizing osteoporosis treatment strategies and ensuring better patient care.

## 1. Introduction

The World Health Organization (WHO) defines osteoporosis as “a systemic skeletal disease characterized by low bone mass, deterioration of bone tissue, and disruption of bone microarchitecture, leading to compromised bone strength and an increase in the risk of fractures [1].” The US National Institutes of Health simply defines it as “a skeletal disease in which the risk of fracture increases due to weakening of bone strength [2].” The WHO estimates that over 200 million people worldwide suffer from osteoporosis, contributing to 8.9 million fractures annually, or approximately one fracture every three seconds [3,4]. Osteoporosis is a major global health issue, particularly among postmenopausal women, due to the associated risk of fractures and increased healthcare burden.

National Health Insurance data indicate that the prevalence of osteoporosis in South Korean adults aged 50 or older is 22.4%, while that of osteopenia is 48%. More than 5000 out of 100,000 people are counted as suffering from osteoporosis in South Korea [5].

From 2018 to 2023, the number of patients treated for osteoporosis steadily increased at an average annual rate of 4.8%, from 9.8 million in 2018 to 11.8 million in 2022. As of 2022, female patients accounted for 94.4% of the total, indicating that the frequency of osteoporosis in women is about 17 times greater than that in men [6]. Along with this trend, the total medical expenses for osteoporosis increased by 51% from 2015 to 2019 (from USD 155 million to USD 234 million), while the medical expenses per patient increased by 3.7% annually, reaching USD 216 [7].

Osteoporosis treatment can be broadly divided into general lifestyle recommendations, which can benefit most postmenopausal women, and specific pharmaceutical treatments [8]. General recommendations include using non-pharmacological strategies such as calcium and vitamin D supplementation, performing appropriate weight-bearing and muscle-strengthening exercises, avoiding smoking and excessive alcohol consumption, and preventing falls [9]. Antiresorptive and osteoanabolic drugs are used as pharmaceutical treatments in patients with osteoporotic fractures and in those with increased fracture risk due to osteoporosis [10].

Since the WHO declared the coronavirus disease (COVID-19) a pandemic in 2020, prolonged restrictions on healthcare accessibility, reduced outdoor activity, and increased indoor living have heightened the risk of vitamin D deficiency in patients with osteoporosis [11,12]. Vitamin D is an essential nutrient that facilitates calcium absorption and regulates bone formation, playing a crucial role in the prevention and treatment of osteoporosis [13]. Notably, during the COVID-19 pandemic, the use of vitamin D supplements increased, and some studies have suggested that vitamin D may exert immunomodulatory effects, potentially offering protective benefits against viral infections [14]. These findings underscore how the importance and consumption of specific treatments can be significantly influenced by changing global circumstances.

Bisphosphonates represent a class of antiresorptive drugs that currently dominate pharmaceutical treatment worldwide, but their use is limited because they only inhibit bone resorption and have been associated with various side effects such as atypical femoral fractures, osteonecrosis of the jaw, and gastrointestinal irritation [15,16]. However, the recent development of denosumab, the most powerful antiresorptive agent, and the osteoanabolic drug romosozumab have increased treatment options for patients with osteoporosis. With the continuous introduction of new medications and changes in usage standards, the trend of use is also changing.

Our primary objective in this study was to analyze the market dynamics of osteoporosis medications in South Korea from 2018 to 2023 using data from Intercontinental Marketing Services (IMS). Specifically, we aimed to assess sales trends, shifts in market share among drug classes, and the impact of newer treatments, such as denosumab and romosozumab, on prescribing patterns. The secondary objectives were to evaluate factors influencing these market trends, including changes in osteoporosis treatment guidelines, reimbursement policies, and the potential impact of external factors such as the COVID-19 pandemic on drug utilization.

## 2. Methods

The list of drugs approved for the treatment of osteoporosis in South Korea in 2023 is provided in Supplementary Table S1 [17]. Among them, the costs of items and drugs sold for osteoporosis prevention and treatment between 2018 and 2023 were calculated based on IMS data (in USD).

Osteoporosis drugs were divided into general treatments (calcium and vitamin D supplementation) and pharmaceutical agents (hormone and non-hormonal therapies). In accordance with IMS recommendations, the analysis of hormone therapy included female hormones (estrogen only or an estrogen–progesterone combination), tibolone, and a tissue-selective estrogen complex (TSEC). Non-hormonal therapies included bisphosphonates; selective estrogen receptor modulators (SERMs); raloxifene and bazedoxifene; calcitonin; parathyroid hormones (PTHs); denosumab, the recently approved receptor activator of nuclear factor-κB ligand (RANKL); and romosozumab, the monoclonal anti-sclerostin antibody. This study was granted an exemption by the Institutional Review Board (IRB) of The Catholic University of Korea, Seoul, St, Mary’s Hospital (IRB No KC21ZASI1011). This study followed the Reporting of Studies Conducted Using Observational Routinely Collected Data (RECORD) guidelines to ensure transparency and reproducibility in reporting pharmaceutical sales data. RECORD provides a structured framework for studies using administrative and healthcare databases. Additionally, the Consolidated Health Economic Evaluation Reporting Standards (CHEERS) checklist was applied to enhance the clarity of the economic analysis. CHEERS ensures the standardized reporting of key elements in health economic evaluations. By adhering to these guidelines, we aimed to improve the methodological rigor and clarity of our findings [18,19].

### Statistical Analysis

To evaluate temporal trends in the annual sales volumes of osteoporosis medications between 2018 and 2023, we conducted a linear trend analysis using simple linear regression. For each medication, the year was treated as a continuous independent variable, and the annual sales volume was treated as the dependent variable. The slope and *p*-value of each regression model were examined to determine the presence of a statistically significant increasing or decreasing trend over time. A *p*-value of less than 0.05 was considered indicative of a significant trend. The coefficient of determination (R^2^) was also reported to evaluate the goodness-of-fit for each model. All statistical analyses were performed using SPSS version 23.0 (IBM Corp., Armonk, NY, USA).

## 3. Results

### 3.1. Market Size for Osteoporosis Medications in Korea in 2023

**Figure 1:**
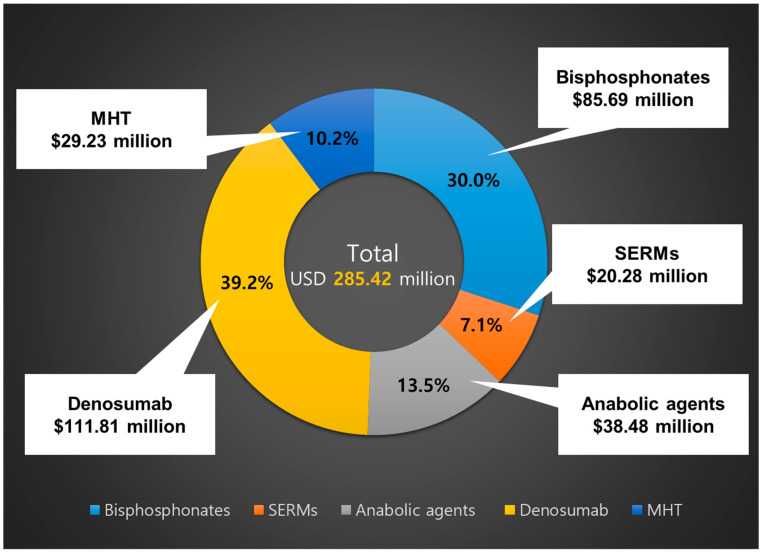
Market size for osteoporosis treatment in Korea in 2023. SERMs, selective estrogen receptor modulators; MHT, menopausal hormone therapy; USD, US dollar.

The total market size of osteoporosis medications in 2023 was USD 285.42 million, which is a 15.3% increase from USD 257.38 million in 2022. Denosumab accounted for 39.2%, followed by bisphosphonates at 30.0% of the total market size (USD 85.69 million), menopausal hormone therapy at 10.2%, SERMs at 7.1%, and anabolic agents at 13.5% (Figure 1). Among pharmaceutical treatments, hormonal and non-hormonal drugs for osteoporosis accounted for 10.2% (USD 30.38 million) and 89.8% (USD 266.31 million) of the total market, respectively.

### 3.2. Hormonal Treatments

**Figure 2:**
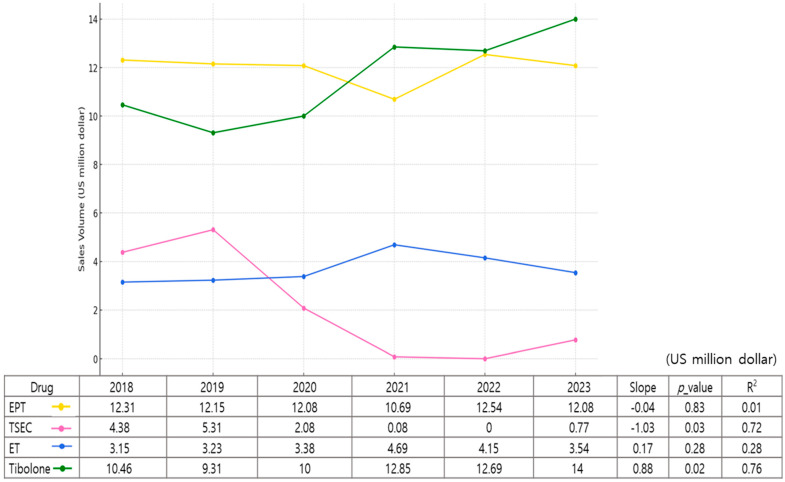
Trends in hormonal treatments for osteoporosis (2018–2023). EPT, estrogen–progestogen therapy; TSEC, tissue-selective estrogen complex; ET, estrogen-only therapy.

Postmenopausal female hormone therapy includes estrogen-only (ET) treatment, estrogen–progestogen therapy (EPT), tibolone, and TSEC. The total cost of hormonal agents used in 2023 was USD 30.38 million, showing no significant change compared to USD 30.31 million in 2018. In 2023, ET and EPT treatments represented 51.4% of the total market share, followed by tibolone at 46.1% and TSEC at approximately 2.5%. A linear trend analysis was conducted to assess annual changes in the sales volumes of four osteoporosis medications from 2018 to 2023. Among the four agents, TSEC showed a statistically significant decreasing trend over the 6-year period (slope = −1.028, *p* = 0.033, R^2^ = 0.718), whereas tibolone exhibited a significant increasing trend (slope = +0.877, *p* = 0.023, R^2^ = 0.764). No statistically significant trend was observed for EPT (slope =−0.039, *p* = 0.833, R^2^ = 0.013) or ET (slope = +0.172, *p* = 0.278, R^2^ = 0.282), although ET demonstrated a slight upward trend over time. (Figure 2).

### 3.3. Non-Hormonal Treatments

**Figure 3:**
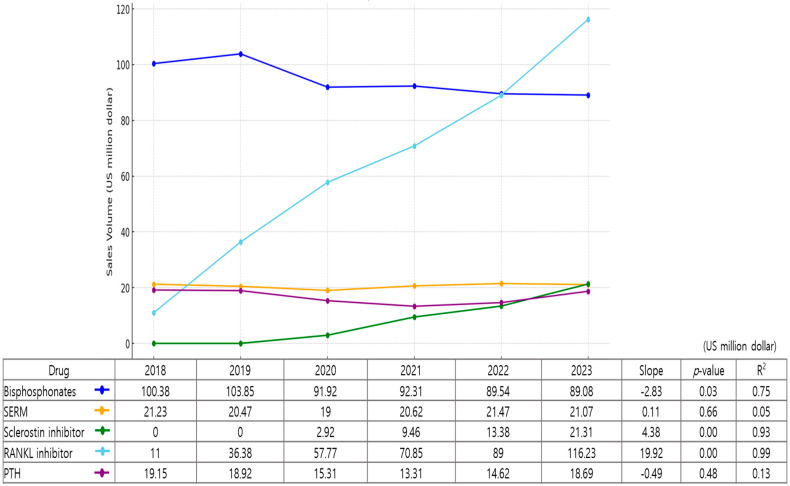
Trends in non-hormonal treatments for osteoporosis (2018–2023). SERM, selective estrogen receptor modulator; RANKL, receptor activator of nuclear factor kappa-B ligand inhibitor; PTH, parathyroid hormone.

Excluding hormonal treatments, the total sales of osteoporosis drugs in 2023 was USD 266.31 million, with bisphosphonates accounting for approximately one-third of the market share (33.4%, USD 89.08 million). Denosumab, a RANKL inhibitor, accounted for the largest share of the market at 43.6% (USD 116.23 million). Sales rose from USD 11 million in 2018 to USD 36.38 million in 2019 and further to USD 116.23 million in 2023, representing a 957.6% increase. In 2023, denosumab experienced a 31.6% increase compared to the previous year and ranked first in sales among single-agent components. SERMs (including bazedoxifene and raloxifene) accounted for 7.1% of the market share (USD 20.28 million), while anabolic agents such as teriparatide and romosozumab accounted for another 13.5% (USD 38.48 million). Romosozumab has been available in the South Korean market since 2020, accounting for 7.4% (USD 21.31 million) of the market share in 2023. (Figure 3).

A linear trend analysis was conducted to evaluate changes in the annual sales volumes of five major osteoporosis medications from 2018 to 2023. Bisphosphonates showed a statistically significant decreasing trend over this period (slope = −2.83, *p* = 0.0262, R^2^ = 0.748), while RANKL inhibitors demonstrated the most substantial increase (slope = +19.92, *p* < 0.0001, R^2^ = 0.990). Sales of sclerostin inhibitors also increased significantly (slope = +4.38, *p* = 0.0021, R^2^ = 0.927), reflecting the emerging role of anabolic agents in osteoporosis treatment. In contrast, there were no statistically significant trends observed for SERMs (slope = +0.11, *p* = 0.6611, R^2^ = 0.053) or PTH analogs (slope =−0.49, *p* = 0.4835, R^2^ = 0.130), suggesting a relatively stable market trend for these agents during the study period.

### 3.4. Changes in Prescription Patterns for Bisphosphonates

The analysis of bisphosphonate usage trends from 2018 to 2023 revealed a decline in the overall market volume, with a 5-year reduction of 11% (100.38 in 2018 to 89.08 in 2023). While oral bisphosphonates, such as alendronate and ibandronate, have shown consistent decreases in their usage over the years (−19% and −25%, respectively), risedronate demonstrated a modest recovery with a 2% increase in 2023 compared to 2022. Intravenous bisphosphonates present a contrasting trend; while zoledronate usage decreased significantly by 36% over five years, intravenous ibandronate stood out with a 20% increase during the same period, and its upward trend was sustained in the most recent year (Table 1).

### 3.5. Global Trends in Osteoporosis Drug Sales

#### Three Year Sales Trends in the United States, Japan, and South Korea (2021–2023)

**Figure 4:**
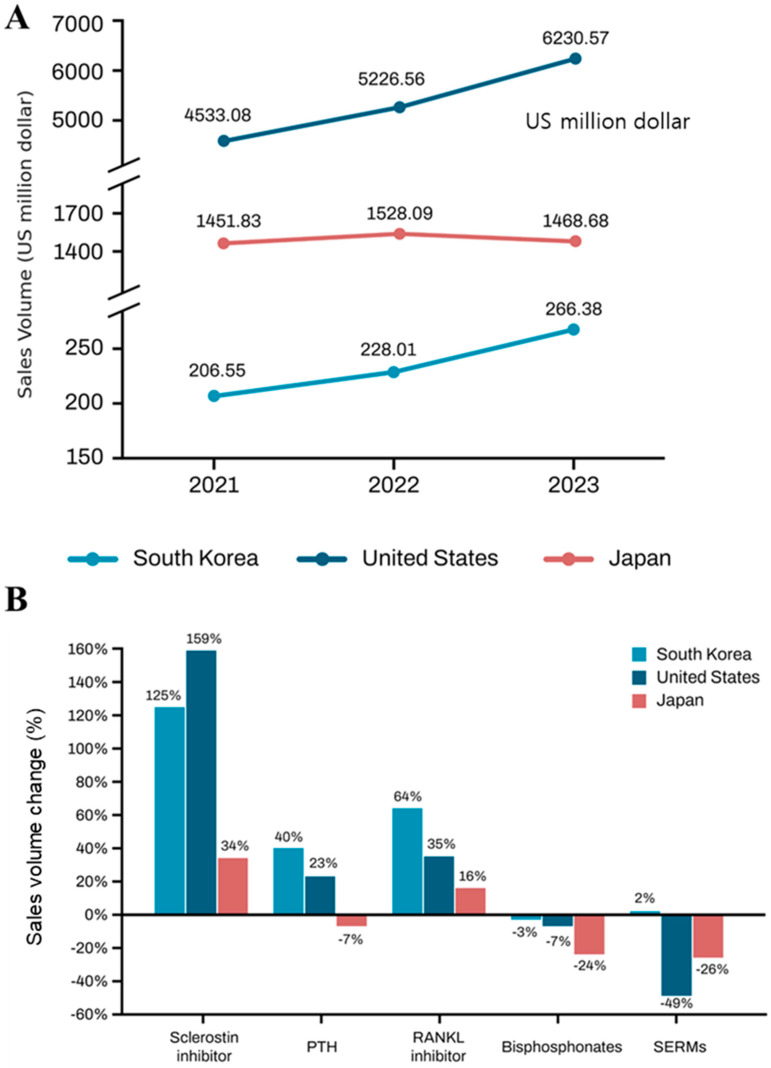
Three-year sales trends in the United States, Japan, and South Korea (2021–2023). (**A**). Total sales of osteoporosis medications over three years in the United States, Japan, and South Korea. (**B**). The growth rate of each osteoporosis medication by country. SERMs, selective estrogen receptor modulator; RANKL, receptor activator of nuclear factor kappa-B ligand inhibitor; PTH, parathyroid hormone.

From 2021 to 2023, the total sales of osteoporosis drugs increased significantly in the United States by 37.45%, highlighting its robust market expansion. This is primarily attributed to the significant growth in RANKL and sclerostin inhibitors. South Korea also experienced a substantial growth rate of 28.97%, reflecting its emerging market potential. In contrast, Japan recorded a modest growth of only 1.16%, indicating a stagnant or mature market with limited expansion during this period. This appears to have been impacted by the decreased use of bisphosphonates and PTH (Figure 4).

## 4. Discussion

Currently, 200 million people worldwide have osteoporosis, with estimates indicating that even more are living with osteopenia [20]. More than 8.9 million fractures are caused by osteoporosis every year, occurring approximately once every 3 s [21]. The number of patients with osteoporotic compression fractures in South Korea increased from 117,361 in 2012 to 139,889 in 2016, and 54.3% of these patients received hospital treatment [22]. Along with the increase in the number of fractures, treatment costs have also increased rapidly [22]. In 2003, the estimated total socio-economic loss associated with osteoporosis in South Korea was USD 871.7 million, with annual medical expenses and loss of productivity costs reaching USD 364.6 million and USD 566.4 million, respectively [22]. Similarly, the osteoporosis drug market also continues to expand, and the seven major markets for osteoporosis treatment in the United States, Japan, and Europe are expected to grow from approximately USD 6.1 billion in 2014 to USD 9.34 billion in 2024 [23]

This study aimed to analyze the size and trends of the osteoporosis drug market in South Korea from 2018 to 2023. According to a previous study that evaluated markets for hormonal and osteoporosis treatments in South Korea from 2016 to 2019, the osteoporosis drug market has expanded from approximately USD 133.4 million in 2016 to USD 210 million in 2019, with bisphosphonates accounting for the largest share and exhibiting a steady increase [24]. In the analysis of hormonal therapies for the prevention and treatment of postmenopausal osteoporosis, the authors noted that sales of TSEC increased annually while EPT sales gradually decreased [24]. However, in the case of TSEC, the product was withdrawn from the market in 2019 due to a defect in the aluminum laminate packaging, which allowed oxygen ingress and subsequently reduced the dissolution rate of the active pharmaceutical ingredient. After quality improvements were made, its supply was resumed in 2023 [25]. From 2019 to 2023, the sharp decline in TSEC sales, accompanied by a gradual increase in tibolone use, resulted in a stagnation in the overall market share of postmenopausal hormonal agents in 2023 compared to 2018.

Denosumab received approval for reimbursement as a second-line treatment for osteoporosis in October 2017 in Korea [26]. Initially, the criteria for the reimbursement of Prolia as a second-line treatment were restrictive, requiring patients to have experienced new fractures or a decrease in their T-score on bone density tests after using bisphosphonates for more than one year [26]. However, in April 2019, the approval was expanded to include denosumab as a first-line treatment, leading to a significant increase in sales [26]. Denosumab is a human monoclonal antibody against RANKL that inhibits differentiation and bone resorption in osteoclasts [27]. Previous clinical studies have highlighted the excellent efficacy and safety of denosumab treatment in reducing fracture incidence in various patient groups [27]. The most important study that demonstrated the efficacy of denosumab in postmenopausal women with osteoporosis was the Fracture Reduction Evaluation of Denosumab in Osteoporosis Every Six Months (FREEDOM) study [28]. Subsequently, the FREEDOM Extension study, which evaluated the efficacy and safety of the drug over 10 years by extending the study period by 7 years, revealed that the incidence of new vertebral fractures was 0.90–1.86%, while the incidence of non-vertebral fractures was 0.84–2.55% [29]. The Determining Efficacy: Comparison of Initiating Denosumab versus Alendronate (DECIDE) study compared the effects of bisphosphonates and denosumab [30]. The denosumab administration group exhibited significantly higher bone densities in the lumbar spine, femoral neck, and femur than the alendronate administration group (with increases of 1.1%, 0.6%, and 1.0%, respectively), indicating that its effects were superior to those of existing drugs [30]. Furthermore, the FREEDOM Extension study reported that the incidence of adverse reactions such as severe infections, cellulitis, eczema, and malignant tumors was low in patients treated with denosumab [29]. In addition, osteonecrosis of the jaw occurred in only 5.2 cases per 10,000 patient years, indicating that denosumab administration does not require an off period during long-term use in contrast to bisphosphonates [29]. Based on the current analysis, the use of denosumab is predicted to increase due to its demonstrated efficacy, low risk of side effects, and the expansion of insurance standards in 2019.

Romosozumab is the latest osteoporosis treatment to be approved by the U.S. FDA (April 2019) [31]. PTH agents, which represent the only anabolic agents approved for osteoporosis prior to April 2019, increase both bone formation and resorption [32]. In contrast, despite being a single drug, romosozumab inhibits bone resorption while promoting bone formation [33]. In the FRActure study in postmenopausal woMen with osteoporosis (FRAME), a phase 3 clinical trial of romosozumab showed vertebral and femoral bone densities to increase by 13.3% and 6.8%, respectively, after one year of treatment with romosozumab when compared to those observed in the placebo group [34].

The STRUCTURE trial compared outcomes for romosozumab treatment with those for treatment with teriparatide, which is the same type of anabolic agent, reporting better results in the romosozumab group, showing a more positive effect on hip bone mineral density in particular [35].

In the Active-Controlled Fracture Study in Postmenopausal Women With Osteoporosis at High Risk (ARCH) study comparing the outcomes of romosozumab and alendronate treatment after 1 year, there was a 2.5% incidence of cardiovascular adverse events in the romosozumab group compared to 1.9% in the alendronate group, even though the incidence of fractures was significantly reduced in the romosozumab group [36]. Hence, the FDA approved the use of romosozumab with the stipulation that it cannot be used in patients who have recently experienced a myocardial infarction or stroke [37]. Recently, osteoporosis treatment groups have been classified according to fracture risk, and the use of anabolic agents is primarily recommended in groups with a very high risk of fracture (e.g., patients with severe osteoporosis accompanied by fractures or multiple vertebral fractures) [38].

In the United States, following FDA approval in April 2019, romosozumab was included in Medicare Part B, the public health insurance system operated by the federal government. Additionally, many major private insurers began covering this medication as a reimbursable treatment starting in 2020 [39]. Through this process, initial reimbursement coverage targeted patients with severe osteoporosis, allowing treatment without prior authorization or step therapy requirements. This increased the acceptance of the new drug among physicians and patients, driving significant growth in the osteoporosis market.

Similarly, in Japan, following its approval in May 2019, the Osteoporosis Fracture Score system was developed to identify high-risk groups for hip fractures and emphasize the importance of proactive early treatment with anabolic agents. Patients diagnosed with osteoporosis continued to receive medical insurance coverage without interruption, as there were no criteria for discontinuing reimbursement, leading to a high proportion of romosozumab usage in treatment [40,41,42].

Romosozumab was approved by the Ministry of Food and Drug Safety in South Korea in June 2020, allowing its use in the country [43,44]. In South Korea, the current insurance coverage criteria limit its use to patients for whom previous anti-osteoporosis therapies have been ineffective or to patients aged 65 and older with a T-score of −2.5 or below and two or more osteoporotic fractures [45]. Although these restrictions have resulted in a relatively low market share of 7.4% for romosozumab, its high therapeutic efficacy has led to a growing trend in usage, with a notable 59% increase in sales in 2023 compared to 2022. In addition, the recently published guidelines from the American Association of Clinical Endocrinologists (AACE) and the American College of Endocrinology (ACE) recommend romosozumab as a first-line treatment for patients at very high risk of fractures, and its use is expected to increase further [38].

In addition to the shift toward newer agents such as denosumab and romosozumab, notable trends have been observed within the bisphosphonate class. Between 2018 and 2023, the overall market volume of bisphosphonates declined by 11%, reflecting a gradual reduction in the use of oral formulations. Specifically, the use of alendronate and oral ibandronate decreased by 19% and 25%, respectively. In contrast, risedronate showed a modest rebound with a 2% increase in 2023 compared to the previous year. Interestingly, intravenous bisphosphonates exhibited divergent trends; while the usage of zoledronate declined sharply by 36% over five years, intravenous ibandronate experienced a 20% increase during the same period, and this upward trend has been sustained in the most recent year. These findings suggest a growing preference for intravenous agents—particularly ibandronate—likely due to its quarterly dosing regimen and lower gastrointestinal side effects, which may enhance patient adherence [46]. This underscores the increasing importance of treatment convenience and tolerability in the evolving landscape of osteoporosis management.

These findings should also be considered in the context of broader social and healthcare system changes. With the onset of the coronavirus infection in China at the end of 2019, COVID-19 in 2020 was classified as a pandemic by the WHO on 11 March 2020. In response, many countries have implemented unprecedented measures to contain the spread of the virus, including nationwide lockdowns, massive social quarantines, restrictions on public gatherings, and travel bans. These social distancing strategies created difficulties in the management of many chronic diseases. It cannot be ruled out that this social and medical environment may have caused changes in drug treatment.

In osteoporosis medication treatment, a critical focus lies in the treat-to-target strategy for reducing fracture risk and implementing national treatment strategies that ensure the continuity of care through changes in the reimbursement criteria. In South Korea, significant changes in medication usage frequency have been noted following modifications to the reimbursement criteria. For medications with restrictive reimbursement criteria, their usage frequency has been observed to be relatively low despite their clinical efficacy.

However, as of May 2024, reimbursement criteria were revised. Previously, osteoporosis medications were reimbursed only for patients with a T-score of −2.5 or lower [47]. The updated criteria now extend reimbursement eligibility to patients with T-scores exceeding −2.5 but not exceeding −2.0, allowing continuous treatment for up to two years. This revision applies to raloxifene, bazedoxifene, bisphosphonates, and denosumab injections, with flexibility for switching between these treatments based on dosage and administration requirements. This expansion ensures the continuity of treatment.

### 4.1. Perspectives for Clinical Practice

The findings of this study provide valuable insights into the evolving landscape of osteoporosis treatment in South Korea and have significant implications for clinical practice. The increasing use of romosozumab and denosumab indicates a shift toward anabolic and potent antiresorptive agents for high-risk patients with osteoporosis. The 2024 reimbursement revision expanded coverage to T-scores between −2.5 and −2.0 and is expected to improve treatment access and long-term outcomes. The decline in oral bisphosphonate use and the preference for injectables highlight the need for treatment adherence and patient-centered care. Clinicians should consider shared decision-making to optimize therapy selection.

The monitoring of real-world effectiveness and long-term outcomes is essential for refining osteoporosis management and healthcare policies.

### 4.2. Limitations

First, although hormonal replacement therapy has been indicated to prevent and treat osteoporosis, it is also used to treat other menopausal symptoms. However, our ability to subdivide and understand these different indications was limited. Second, since the market size was determined based only on the total sales, there may be a difference between the actual numbers of prescriptions or agents used. Nonetheless, most drugs, including hormonal replacement therapy, have been analyzed, making it easy to evaluate the overall drug market. Third, the sales trends do not directly capture patient adherence, treatment persistence, or clinical outcomes. Fourth, this study does not quantitatively assess the long-term impact of the COVID-19 pandemic on osteoporosis management. Future research incorporating real-world evidence studies, long-term treatment outcomes, and international comparisons are necessary to refine osteoporosis treatment strategies and improve patient care. Despite these limitations, this study provides critical insights into the evolving osteoporosis drug market and serves as a foundation for future research.

## 5. Conclusions

This study highlights key trends in the osteoporosis drug market in South Korea from 2018 to 2023, including the increasing use of anabolic agents, such as romosozumab and denosumab, and the impact of the 2024 reimbursement revision, which expands treatment access for patients with T-scores between −2.5 and −2.0.

The shift from oral bisphosphonates to injectable therapies emphasizes the need for patient-centered treatment approaches that improve adherence and convenience. While this study provides valuable market insights, it does not capture patient adherence, treatment persistence, or long-term clinical outcomes.

Future research should incorporate real-world evidence and long-term effectiveness studies to further refine osteoporosis treatment strategies and healthcare policies.

## Figures and Tables

**Table 1:** Evolving prescription trends for oral and intravenous bisphosphonates.

Category	Therapy	2018	2022	2023	1-Year Growth Rate (2022 vs. 2023)	5-Year Growth Rate (2018 vs. 2023)
Oral BP agent	Alendronate	25.15	20.92	20.46	−2%	−19%
	Risedronate	26.08	19.85	20.31	2%	−22%
	Ibandronate	11.77	9.08	8.85	−3%	−25%
IV BP agent	Zoledronate	9.69	6.92	6.23	−10%	−36%
	Ibandronate	27.69	32.77	33.23	1%	20%
Total Bisphosphonates		100.38	89.54	89.08	−1%	−11%

(US million dollar). BP, bisphosphonates; IV, intravenous.

## Data Availability

The datasets generated during and/or analyzed in the current study are available from the corresponding author upon reasonable request.

## Supplementary Materials

The following supporting information can be downloaded at: https://www.mdpi.com/article/10.3390/medicina61050805/s1, Table S1: Types of osteoporosis treatment.
