# Supplementary material for: Five-Year Sales Trends of Osteoporosis Medications in Korea: A Market Analysis Based on IMS Health Sales Audit Data (2018–2023)
Source: Medicina (Kaunas). 2025 Apr 26;61(5):805. doi: 10.3390/medicina61050805 (PMC12112770; doi:10.3390/medicina61050805)
Supplement: Supplementary file 1 [file medicina-61-00805-s001.zip › medicina-3488926-supplementary.pdf]

Supplementary Table S1. Types of osteoporosis treatment.

Antiresorptive agents	Therapy	Dose	Schedule and route
**Bisphosphonates**	Alendronate	10 mg	PO once a day
		70 mg	PO once a week
	Alendronate + Cholecalciferol	70 mg + 2800 IU	PO once a day
	Alendronate + Calcitriol	5 mg + 0.5 µg	PO once a day
	Risedronate	5 mg	PO once a day
		35 mg	PO once a week
		35 mg enteric-coated	PO once a day
		75 mg	PO 1 month for 2 days
		150 mg	PO once a month
	Risedronate + cholecalciferol	35 mg + 5600 IU	PO once a week
		150 mg+ 30000 IU	PO once a week
	Pamidronate	30 mg	IV once every 3 months
	Ibandronate	150 mg	PO once a month
		3 mg	IV once every 3 months
	Ibandronate + cholecalciferol	150 mg + 24000 IU	IV once a month
	zoledronic acid	5mg	IV once a year
**Hormonal treatments**	Estrogen ± Progestogen	Differed according to type	PO once a day<br>Once a day estrogen gel
	Tibolone	2.5 mg	PO once a day

**TSEC**	Bazedoxifene + conjugated estrogen	20 mg + 0.45 mg	PO once a day, approved for the prevention of osteoporosis regardless of meal
**SERMs**	Raloxifene	60 mg	PO once a day
	Raloxifene + Cholecalciferol	60 mg+800 IU	PO once a day
	Bazedoxifene	20 mg	PO once a day
	Bazedoxifene+Cholecalciferol	20 mg + 800 IU	PO once a day
**RANKL inhibitor**	Denosumab	60 mg	Subcutaneous injection once every 6 months

**Anabolic agents**	**Therapy**	**Dose**	**Schedule**
**Parathyroid hormone**	Teriparatide	20 μg	Subcutaneous injection once a day
	Teriparatide acetate	56.5 μg	Subcutaneous injection once a week
**PTHrP analog**	Abaloparatide	80 μg	Subcutaneous injection once a day
**Sclerostin inhibitor**	Romosozumab	210 mg	Subcutaneous injection once a month

**Other**	**Therapy**	**Dose**	**Schedule and route**
**Vitamin D3 derivative**	Calcitriol	0.25μg	PO
	Alfacalcidol	0.5 μg	PO
**Calcitonin**	Salmon calcitonin	200 IU	Intranasal spray once a day

	Elcaltonin	20 IU	IM once a week

TSEC; Tissue Selective Estrogen Complex: SERMs; selective estrogen receptor modulator: RANKL; Receptor activator of nuclear factor-κB ligand: PTHrP; Parathyroid hormone-related protein; IU: International Unit.
